# The impact of horizontal gene transfer on the adaptive ability of the human oral microbiome

**DOI:** 10.3389/fcimb.2014.00124

**Published:** 2014-09-08

**Authors:** Adam P. Roberts, Jens Kreth

**Affiliations:** ^1^Department of Microbial Diseases, UCL Eastman Dental Institute, University College LondonLondon, UK; ^2^Department of Microbiology and Immunology, College of Medicine, University of Oklahoma Health Sciences CenterOklahoma City, OK, USA

**Keywords:** horizontal gene transfer, mobile genetic elements, conjugation, transformation, hydrogen peroxide, extracellular DNA, oral cavity, biofilm

## Abstract

The oral microbiome is composed of a multitude of different species of bacteria, each capable of occupying one or more of the many different niches found within the human oral cavity. This community exhibits many types of complex interactions which enable it to colonize and rapidly respond to changes in the environment in which they live. One of these interactions is the transfer, or acquisition, of DNA within this environment, either from co-resident bacterial species or from exogenous sources. Horizontal gene transfer in the oral cavity gives some of the resident bacteria the opportunity to sample a truly enormous metagenome affording them considerable adaptive potential which may be key to survival in such a varying environment. In this review the underlying mechanisms of HGT are discussed in relation to the oral microbiome with numerous examples described where the direct acquisition of exogenous DNA has contributed to the fitness of the bacterial host within the human oral cavity.

## Introduction

The human oral microbiome is an incredible example of a species rich collection of micro-organisms living together primarily as a multispecies biofilm. The constant challenges the biofilm inhabitants have to cope with include interactions of co-operation and antagonism whilst the individual cells have to adjust to an ever changing onslaught of environmental perturbations. Availability of carbohydrate sources, temperature changes and the interaction with transient, non-oral species of bacteria are just a few examples of the challenges the individual members of the multispecies oral biofilm have to adjust to.

There are many recent reviews concerning the actual numbers, and species composition, of bacteria within the human oral cavity and the reader is directed to these (e.g., Curtis et al., 2011; Wade, 2013), and accompanying articles in this special issue for the background knowledge on the composition of this community.

The dynamic environment of the oral cavity pressures cells of the oral biofilm to not only adjust at the metabolic level but also evolve their genomic content and potential. The species richness and diversity of the oral cavity creates complex bacterial interactions including the exchange of genetic material via horizontal gene transfer (HGT). A recent study on the genome evolution of the genus *Streptococcus*, which is the most abundant genus in the oral cavity (Rosan and Lamont, 2000; Diaz et al., 2006), demonstrated HGT as important mechanism for the acquisition of new genetic traits and significantly contributed to the genomic expansion and streamlining of *Streptococcus* (Richards et al., 2014). Indeed Smillie *et al* have recently shown that the driver for HGT between bacteria is primarily due to the ecology with most gene transfers (determined where genes are >99% identical at the nucleotide level in different bacteria) occurring in bacteria from ecologically similar environments (Smillie et al., 2011) and is less dependent on geography or phylogeny of the microbial community. This makes strategic sense for the organisms which inhabit these different ecological niches as the available, accessory metagenome will be enriched for genes which allow adaption to local stresses and maximization of opportunities within a particular environment. Each environment will have an individual, and often unique, set of parameters which must be tolerated and exploited by the microbial inhabitants in order to successfully colonize that environment over time.

## The variable oral environment

The oral cavity is by no means a static environment; rather it is an environment where diverse ecological pressures exist. As a portal to the distal part of the digestive tract the oral cavity is open to the environment and also has a variety of foods (substrates) pass through it. There is therefore a great deal of variability encountered in terms of physical, chemical and physicochemical characteristics.

Bacteria will have to cope with multiple defense mechanisms within the oral cavity including, but not limited to the production of host antimicrobial compounds such as lactoperoxidase and lysozyme, bacterially derived antimicrobials and bacteriocins, production of immunoglobulins A, G, and M, mucus layers on mucosal surfaces and the constant shedding of epithelial cells. There are also relatively strong mechanical forces which result during chewing, talking and the movement of the tongue. Forces up to 150 Newtons (N) are generated whilst chewing foods such as meat whilst the maximal biting forces have been estimated to be between 500 and 700 N (Wilson, 2005). In addition to the mechanical forces there are also hydrodynamic shear forces that occur due to the flow of saliva and, to a lesser extent gingival crevicular fluid.

Chemically the mouth is a very diverse environment which can be subject to extremely rapid change when food and liquids are consumed. Whilst the main source of nutrients for oral bacteria is the saliva there is a diverse range of carbohydrates and other sources of energy which can be temporarily elevated following feeding and the ability to utilize these substrates rapidly provides an advantage for the microbes. Additionally with so many different species inhabiting the oral cavity, inevitable syntrophic relationships have evolved such as species of *Veillonella* utilizing the lactate produced by cariogenic streptococci (Chalmers et al., 2008).

Differences in mechanical force, nutritional variation and availability, temperature, pH levels, oxidative stress and redox potential, presence of both host and bacterially derived antibacterial enzymes all provide challenges to the microbial inhabitants which inevitably select for evolved advantageous traits. When such traits are encoded by mobile DNA this environment will also therefore select for the transfer of such genes to other inhabitants.

## Horizontal gene transfer in the oral cavity

Conjugation, transduction and transformation are the three main mechanisms of HGT. Conjugation is the direct transfer between live donor and recipient cells in a DNase insensitive manner and is the mechanisms of transfer used by conjugative plasmids and conjugative transposons. Transduction is the transfer of host genomic DNA by bacteriophage which package the host DNA into the bacteriophage head structures and transformation is the uptake of exogenous, extracellular DNA often released from dead bacteria cells in the environment. The mechanics of these three mechanisms have been reviewed in detail previously for specific pathogens (e.g., Lindsay, 2014). Recently however another process involved in HGT has been reported which deserves mention here as it could be directly relevant to the biofilm way of life.

Membrane vesicles are released from the cell surface by many Gram-negative, and some Gram-positive, bacteria and can contain proteins, polysaccharides and importantly for microbial adaptation, DNA (Yaron et al., 2000). This DNA can be utilized by other competent bacteria as a substrate for transformation. Virulence genes, plasmid located antibiotic resistance genes and *gfp* (encoding green fluorescent protein) have been shown to be exported from *Escherichia coli* in vesicles and furthermore have been shown to successfully transform *Salmonella* (Yaron et al., 2000; Mashburn-Warren and Whiteley, 2006). It has been revealed that a small proportion of the membrane vesicles from the psychrotropic bacterium *Shewanella* have a double membrane and therefore contain cytoplasmic contents, providing a much needed explanation of how DNA can be incorporated into the vesicles without being either transported to the periplasm of the cell or to the external environment and subsequently integrated into a vesicle composed only of the outer membrane (Pérez-Cruz et al., 2013). These DNA containing membrane vesicles have also recently been shown for *Acinetobacter baylyi* (Fulsundar et al., 2014). More recently, the oral biofilm relevant *Streptococcus mutans* has been shown to release extracellular DNA (eDNA) via membrane vesicles into the developing biofilm and provides therefore an important source for genetic material via this novel mechanism (Liao et al., 2014).

HGT has been demonstrated to occur between a wide range of bacteria which inhabit the human oral cavity. Using *in vitro* models both conjugation and transformation have been shown to occur between different species of bacteria (Roberts et al., 1999, 2001; Ready et al., 2006; Hannan et al., 2010) and acquisition of doxycycline resistance encoding transposons has also been demonstrated within a patient receiving doxycycline therapy for the treatment of periodontitis (Warburton et al., 2007). Importantly, gene transfer from transient bacteria unable to colonize oral biofilms themselves has been previously demonstrated to occur from a *Bacillus subtilis* donor of the conjugative transposon Tn*5397* to an oral *Streptococcus* sp. in a mixed species oral biofilm growing in a constant depth film fermentor (Roberts et al., 1999). Additionally bacterial DNA from transient species has been detected in metagenomic libraries made from DNA isolated from the pooled saliva of 20 healthy individuals (Seville et al., 2009); in this case the likely source of the cloned DNA was from a transient *Phytoplasma* sp., which usually resides within plant tissues. This work demonstrates that the plasticity of the oral metagenome may be influenced by the members of transient bacteria which interact with the oral community and will depend themselves on the diet and habits of the individual human host.

Whilst there have been no reported observations of transduction in the oral cavity, or relevant *in vitro* models, there is now good evidence that bacteriophages are abundant in the oral cavity. Studies on saliva have isolated bacteriophages able to lysogenize specific bacterial pathogens such as *Enterococcus faecalis* (Bachrach et al., 2003; Stevens et al., 2009) and *Aggregatibacter actinomycetemcomitans* (Sandmeier et al., 1995; Willi et al., 1997). More recently direct observation and metagenomic analysis of viral plaque and saliva fractions have revealed that bacteriophages are extremely common (Al-Jarbou, 2012; Pride et al., 2012). It has been determined that viral particles are present at approximately 10^8^ particles per milliliter of saliva and that the vast majority of these particles are bacteriophages that may be a reservoir of genes involved in pathogenicity (Pride et al., 2012).

Recent reviews have summarized examples of HGT within and between species which inhabit the human oral cavity (Roberts and Mullany, 2006, 2010; Olsen et al., 2013) however some individual examples pertaining to life in the oral cavity will be discussed below.

## Adherence and biofilm formation

Many, if not most bacteria which inhabit the oral cavity grow as a biofilm on the non-shedding surfaces of the teeth. Therefore the ability to adhere to the oral surfaces and to grow within a biofilm will give these bacteria a significant advantage in this environment due to the advantages of the biofilm mode of growth, many of which are discussed above. One recent example demonstrating the extent to which HGT can allow an organism to survive, and cause disease in the oral cavity is the recently published genome and transcriptome of *Streptococcus parasanguinis* FW213 (Geng et al., 2012). The genome of this bacterium contains at least five acquired genomic islands (GIs). The first two GIs, Fwisland_1 and Fwisland_2 contain genes which are likely to be involved in the production of the lantibiotic salivaricin B and a lactococcin 972 type bacteriocin respectively. The production of bacteriocins and other bacterial inhibitors, particularly by the oral streptococci, which usually are active against similar bacteria to the producing strain, gives them a clear advantage during growth in a relatively nutrient limited environment such as the oral cavity (see below). The third GI, Fwisland_3 encodes long fimbriae that are involved in enhanced biofilm formation. Fwisland_4 encodes genes whose predicted products are involved with the biogenesis and export of extracellular polysaccharides whose roles in biofilm formation, adherence and resistance to host immune systems, such as phagocytosis are well known. Finally Fwisland_5 encodes genes which are believed to be involved in the modulation of biofilm formation. All of the predicted function of the genes products from the 5 GIs appear to be involved in adaptation to the oral cavity (Geng et al., 2012). A similar contribution of HGT to the genomes of other oral species have also been demonstrated, e.g., *Porphyromonas gingivalis* (Tribble et al., 2007, 2012; Kerr et al., 2014) and the mitis group streptococci (Zähner et al., 2011). The ability of bacteria to grow as a biofilm within the oral cavity can also protect them from exogenous antibacterial compounds such as disinfectants and antibiotics. Another way HGT can contribute to the survival under antibiotic pressure is to allow the bacteria to acquire specific genes encoding antibiotic resistance proteins which will be discussed in the next section.

## Acquired antibiotic resistance and mobile genetic elements

One of the most prominent phenotypic advantages a bacterial cell can exhibit is resistance to antimicrobial compounds which are present in great abundance in the oral environment. Acquired antibiotic resistance genes have been found in many species of bacteria which inhabit the oral cavity (Ciric et al., 2012) and additionally recent studies have focussed on metagenomes from the oral cavity (saliva) and have shown a myriad of different resistance genes being present (Seville et al., 2009). Often these resistance genes have been found to be localized on putative and sometimes proven mobile genetic elements (MGEs) (e.g. Ciric et al., 2011, 2014). The conjugative transposon Tn*916* is a prime example of a MGE which is found to be responsible for the transfer of a multitude of different resistances in the oral microflora.

Tn*916* normally confers tetracycline and minocycline resistance by encoding for the Tet(M) protein, a ribosomal protection protein which reversibly binds to the 23S rRNA subunit of the ribosome and prevents tetracycline binding therefore preventing protein synthesis, or removing a bound tetracycline molecule before binding itself (Connell et al., 2003). Tn*916* is the paradigm of a large family of MGEs, many of which encode additional resistance genes e.g. elements such as Tn*2009* contain *erm*(B) conferring macrolide, lincosamide and streptogramin resistance, Tn*6009* encodes resistance to both inorganic and organic mercury by the action of MerA and MerB respectively and Tn*1545* and Tn*6003* both encode resistance to kanamycin via the product of *aphA*-3 (reviewed in Roberts and Mullany, 2011). Interestingly the first antiseptic resistance gene has recently been found associated with a Tn*916* like element designated Tn*6078*. This antiseptic resistance gene; *qrg*, is itself flanked by two copies of a commonly found insertion sequence IS*1216*. This composite transposon has inserted into a gene encoding a protein Orf15 which is believed to be essential in the conjugation of the host Tn*916*-like element. Experimentation failed to demonstrate transfer by conjugation of this element, presumably due to the insertion in *orf15*, however it was demonstrated that it could successfully transform a competent oral streptococci (Ciric et al., 2011) to CTAB resistance. This study is one of a number to highlight the redundancy in the mechanisms of HGT of resistance genes in this environment and it shows that both conjugation and transformation are important in terms of antimicrobial resistance transfer (Hannan et al., 2010). The regulation of competence and uptake of naked DNA in the oral environment is pervasive and is perhaps the driving force for adaptation in some species of oral bacteria such as the streptococci. Most of the previously mentioned MGEs with their different resistances have recently been found in a survey of oral streptococci from 20 healthy adult UK volunteers who provided saliva (Ciric et al., 2012) demonstrating how common these elements, and resistances, are in the general population. Similarly two recent surveys of plasmids from endodontic derived enterococci identified a large number of different replicons containing a larger number of different resistance genes (Song et al., 2013; Wardal et al., 2013).

## Metabolic adaptability

An interesting example of the influence of HGT on the metabolic capability is provided by the lactobacilli. Mammalian associated lactobacilli are common in the human gastrointestinal tract and in the oral cavity. A recent report has described the catabolic versatility of the different species found in this environment and shown that the ability to use dietary carbohydrates is commonly associated with the acquisition of a particular plasmid encoding the relevant metabolic pathways (O'Donnell et al., 2013). One interesting and well characterized strain is *Lactobacillus salivarius* UCC118 which was originally isolated from the terminal ileum of a patient undergoing reconstructive surgery on their urinary tract (Claesson et al., 2006). This strain contains a 242 kb megaplasmid designated pMP118 and two cryptic plasmids (Li et al., 2007; O'Donnell et al., 2013). The pMP118 megaplasmid contains, among others, genes predicted to encode proteins involved in pentose and polyol utilization and genes involved in glycolysis making this particular plasmid extremely beneficial to the carrying strains in carbohydrate rich environments such as the oral cavity and GI tract.

## Horizontal gene transfer and extracellular DNA

The molecular mechanisms of HGT have been investigated in great detail (Frost et al., 2005; Thomas and Nielsen, 2005). While the source of DNA for HGT through mechanisms like conjugation and transduction is obvious, the generation of DNA in the oral biofilm for transformation of competent bacteria is not well understood. In general, DNA for transformation has to be extracellular DNA accessible for competent bacteria. A regulatory relationship between competence development and the generation of eDNA has been shown for pneumococci *in vitro* (Steinmoen et al., 2002; Moscoso and Claverys, 2004), where the release of chromosomal DNA is part of a lytic process controlled by the competence system, termed fratricide (Claverys et al., 2007). The regulatory coordination of DNA release and competence development ensures that the population wide decision to direct energy toward competence development is rewarded by providing the substrate for uptake at the same time. Taking in consideration that an initial clonal population would diverge due to mutation, the extracellular DNA generated during competence development would have enough diversity (mutations) that could favor establishment of new phenotypic traits under the right selective pressure (Luria and Delbrück, 1943). The homologous extracellular DNA released by a competent population of pneumococcus would facilitate easy integration via homologous recombination. The homologous extracellular DNA, however, might pose an evolutionary disadvantage by not providing complex diversity, e.g., genes for new or alternative metabolic pathways or antimicrobial resistance genes as discussed above. Therefore, diversity is most likely achieved by incorporating heterologous DNA from species that are ecologically similar and not of clonal origin (Smillie et al., 2011). This diversity is actually provided in human associated bacterial communities, such as the oral biofilm.

## Extracellular DNA in the oral cavity

A prominent genus of the oral biofilm is *Streptococcus* and competence development is wide spread among this genus (Rosan and Lamont, 2000; Martin et al., 2006; Havarstein, 2010). The biofilm environment of oral streptococci creates a special situation due to the high cell density and species richness (Kreth et al., 2009). Several seminal findings show that oral streptococci are able to co-aggregate with other bacterial species to built up the mature oral biofilm community, which is one of the most diverse human associated communities identified so far (Valm et al., 2011 and references in Kolenbrander et al., 2006). This diversity does not only create cooperation, but also fierce competition (Kreth et al., 2011). Although, the release of eDNA is most-likely mechanistically explained by bacterial lysis, the molecular details and motivation might be diverse.

## Experimentally confirmed mechanisms for eDNA release

### Bacteriocin dependent eDNA release

That bacterial competition causes the release of DNA has been shown (Kreth et al., 2005a; Johnsborg et al., 2008). Initial investigations between the clinically relevant antagonism of *S. mutans* and oral commensal *S. gordonii* have revealed an interesting mechanism of eDNA release. The molecular basis for this antagonism is dependent on bacteriocin production (Kreth et al., 2005a). Bacteriocins are antimicrobial peptides inhibitory toward competing bacterial species. *S. mutans* produces a wide array of bacteriocins and several are regulated by the genetic competence system (Merritt and Qi, 2012). The regulation is due to a specific binding site for the ComE transcriptional regulator found in the promoter sequence of several bacteriocins (van der Ploeg, 2005; Kreth et al., 2006). Once ComE becomes phosphorylated during the activation of the competence cascade, it can bind to the respective bacteriocin gene promoters and activate transcription, while also activating the genes responsible for DNA uptake and homologous recombination. *S. mutans* therefore coordinates its bacteriocin production with competence development (Kreth et al., 2005a), reminiscent of what has been shown for *S. pneumoniae*. The significant difference is that the production of these competence-regulated bacteriocins causes the release of DNA from surrounding competitors, not from itself. Dual species, *in vitro* culture experiments with *S. mutans* and *S. gordonii* have confirmed that induction of the competence system with *S. mutans* specific competence stimulating peptide can result in the release and transfer of transforming DNA from *S. gordonii* to *S. mutans* (Kreth et al., 2005a). Interestingly, the oral bacterial species most susceptible to the bacteriocins under the control of the competence system (namely mutacin IV, V, VI, and Smb) are closely related to *S. mutans* (Merritt and Qi, 2012). This seems to be an ideal mechanism to ensure the availability of DNA with some evolutionary distance but close enough for a high chance of chromosomal integration via homologous recombination. In a recent review about mutacins of *S. mutans*, Merritt and Qi explain the potential ecological role of the coordinated competence and mutacin regulation. *S. mutans* is not an early colonizer and therefore has to face stiff competition from species like *S. sanguinis* and *S. gordonii*, which are abundant species during early biofilm development. By producing mutacins controlled by the competence system, *S. mutans* can eliminate the competition potentially freeing occupied space for its own colonization. The released DNA from the closely related species can easily be taken up and has a high potential to provide new genotypic traits. Conversely, other non-related early colonizers like *Actinomyces* are not targeted by competence-regulated mutacins (Merritt and Qi, 2012). It is tempting to speculate that this is due to the low chance for integration of DNA from distant species into the streptococcal chromosome. In addition, uptake of extracellular DNA released by the action of competence-regulated mutacins might be mainly for homologous recombination and not as a food source or for the generation of DNA building blocks. Competence development in streptococci might in general induce bacteriocins or lytic enzymes to cause lysis of evolutionary related species, as discussed in the next section, to serve in the acquisition of new genetic traits or for DNA repair.

### Murein hydrolase dependent eDNA release

The importance of competence and HGT in its ecological context becomes evident when we revisit the aforementioned pneumococcal fratricide. Initially investigated only in pure single species cultures, fratricide seemed to be an event directed toward its own kind (Claverys et al., 2007). However, in a recent study Johnsborg *et al* demonstrated that fratricide has a broader ecological impact (Johnsborg et al., 2008). *S. pneumoniae* resides in a niche, the oral cavity and nasopharynx that is also inhabited by closely related streptococci like *S. mitis* and *S. oralis* (Dewhirst et al., 2010). Therefore it is not surprising that pneumococcal competence regulated fratricide and the release of DNA affects nearby species. The center of the pneumococcal fratricide is the murein hydrolase CbpD (Wei and Havarstein, 2012). In a concerted action of the autolytic enzymes LytA, LytC and CbpD target cells are lysed to release DNA (Eldholm et al., 2009). While *lytA* and *lytC* are constitutively expressed with an increase of *lytA* expression during competence development, *cbpD* is only expressed in competent cells (Johnsborg et al., 2008). Co-cultivation of *S. pneumoniae* with the closely related *S. mitis* or *S. oralis* demonstrated that CbpD is required for cross species lysis and deletion of CbpD abolished the ability. Further investigation demonstrated that competence induced cell lysis significantly increases HGT between species possessing the CbpD lysis mechanism (Johnsborg et al., 2008; Eldholm et al., 2010). What is the ecological implication? Competence is regulated by a small peptide, termed CSP (competence stimulating peptide), which is secreted and accumulates in the extracellular environment to trigger competence development in a quorum sensing dependent way (Johnsborg and Havarstein, 2009). The CSP sequence is strain and species specific (Johnsborg et al., 2007). In the nasopharynx and oral cavity a diverse number of different CSP pherogroups exist. Once one pherogroup starts to develop competence it also produces the ComM protein rendering the cells immune to the muralytic attack of CbpD. Other pherotypes not developing competence at the same pace, for example due to a lower initial density can now be attacked and the extracellular DNA released during the attack can be taken up by the competent community (Johnsborg et al., 2008; Eldholm et al., 2010). Interestingly, in a follow up study it was shown that the muralytic activity of CbpD at the target cells is dependent on its functional binding to choline-decorated teichoic acids. This limits cross-species attack to streptococcal species with choline-decorated teichoic acids such as *S. mitis* and *S. oralis* (Eldholm et al., 2010). Incidentally, *S. mitis* and *S. pneumoniae* both belong to the mitis group of streptococci and homologous recombination has been discussed as a major driving force for their evolution reflected by a mosaic structure in several gene sequences (Kilian et al., 2008).

### Self-acting intracellular bacteriocin dependent eDNA release

While the action of CbpD can be directed toward itself and other species dependent on the competence state of the respective species, *S. mutans* has a dedicated bacteriocin for intracellular action against itself (Perry et al., 2009). The production of this bacteriocin, termed CipB or mutacin V, is also under the control of the competence system and is stress induced. CipB activity against itself is due to intracellular accumulation in the producer, therefore strictly autolytic. In general, stress situations like low antibiotic concentrations, high cell density and oxygen can induce the competence pathway (Claverys et al., 2006). It is believed that competence and lysis would allow the exchange of fitness-enhancing DNA under those stress conditions (Perry et al., 2009). Since it was shown that the CipB induced lysis only occurs in a sub fraction of cells a valid question is how beneficial the extracellular DNA can be? It is well established that antibiotics can cause DNA damage (Cheng et al., 2013; Dwyer et al., 2014). However, this requires active metabolism and DNA replication. If DNA is released into the environment, the mutagenic potential of certain antibiotics is not working. Hence, cells with damaged DNA could take up the released DNA from its own kind for repair. Obviously this works also in the other direction; if DNA mutation happened due to the presence of antibiotic stress, cells could benefit from the mutated and released DNA, if selection pressure establishes the incorporated DNA as fitness enhancing.

The complexity of bacteriocin production and regulation is best understood in *S. mutans* (Merritt and Qi, 2012). However, the cross-species attack in addition to the internal action of CipB raises the question about the coordination of bacteriocin production. Several mutations in other global regulatory proteins abolish bacteriocin production indicating that the network controlling bacteriocin production is intricate (Chong et al., 2008; Okinaga et al., 2010; Xie et al., 2010). The fluctuating environment of *S. mutans* makes it certain that there are yet to be identified regulatory mechanisms fine tuning bacteriocins production, competence and transformation dependent on environmental signals. In addition, it is not entirely clear when the bacteriocins are produced in the ecological context *in vivo*. Surprisingly, there is a great diversity of bacteriocins produced by different *S. mutans* strains, but all investigated strains produce bacteriocins indicating the importance of this mechanism to ensure the availability of extracellular DNA (Merritt and Qi, 2012).

### H_2_O_2_ dependent eDNA release

A very different mechanism of extracellular DNA generation is used by commensal streptococci *S. gordonii* and *S. sanguinis* (Kreth et al., 2008). Initial investigations on the dual species antagonism with *S. mutans* concentrated on bacteriocin production (Kreth et al., 2005a,b). But the commensals are not the mere victims of this antagonism. *S. sanguinis* and *S. gordonii* can inhibit *S. mutans* in a very efficient way using hydrogen peroxide (H_2_O_2_), and compared to the commensals itself, *S. mutans* is highly H_2_O_2_ susceptible (Kreth et al., 2008). Interestingly, the production of H_2_O_2_ is intimately correlated to the release of DNA. The enzyme responsible for the production of H_2_O_2_ in *S. sanguinis* and *S. gordonii* was identified as pyruvate oxidase or SpxB. SpxB is an oxido-reductase that catalyzes the conversion of pyruvate, inorganic phosphate (Pi) and molecular oxygen (O_2_) to H_2_O_2_, carbon dioxide (CO_2_) and the high-energy phosphoryl group donor acetyl phosphate in an aerobic environment. Knock-out studies with putative pyruvate oxidase orthologs in *S. sanguinis* and *S. gordonii* confirmed SpxB as main H_2_O_2_ producer (Kreth et al., 2008). However, the production of growth inhibiting amounts of H_2_O_2_ is not exclusive to the pyruvate oxidase. Mutational studies with *Streptococcus oligofermentans* showed that at least two other enzymes in addition to the pyruvate oxidase are capable of producing growth-inhibiting amounts of H_2_O_2_. The lactate oxidase LctO catalyzes the formation of pyruvate and H_2_O_2_ from L-lactate and oxygen and an L-amino acid oxidase generates H_2_O_2_ from amino acids and peptones. Antagonism assays of *S. oligofermentans* and *S. mutans* grown in dual species biofilms demonstrated that LctO dependent H_2_O_2_ production is sufficient to antagonize *S. mutans* in an SpxB mutant. The role of the L-amino acid oxidase in interspecies competition is not clear since its inhibiting activity is only visible in a *lctO/spxB* double knock-out mutant (Tong et al., 2007, 2008; Liu et al., 2012). In general, SpxB is a very conserved enzyme encoded by several oral streptococci, including the aforementioned streptococci as well as *S. oralis, S. mitis* as abundant members of the oral biofilm (Zhu and Kreth, 2012; Zhu et al., 2014). Expression of *spxB* in the oral biofilm has recently been confirmed pointing to its active biological function during biofilm development (Zhu et al., 2014). The release of chromosomal DNA into the environment by *S. gordonii* and *S. sanguinis* is closely associated with the production of H_2_O_2_ and an SpxB deletion affected the release significantly (Kreth et al., 2008). In agreement with a diminished production of H_2_O_2_ under anaerobic conditions, a significant reduced concentration of extracellular DNA was detected under oxygen limited growth conditions (Itzek et al., 2011). Interestingly, addition of H_2_O_2_ to anaerobically grown cells does induce DNA release. The H_2_O_2_ induced release process is not entirely understood. An obvious time delay was observed between the addition of H_2_O_2_ and the first detectable amounts of extracellular DNA, suggesting that H_2_O_2_ does not induce immediate lysis of the bacterial cells. No release was observed when cells were treated with chloramphenicol, a known protein biosynthesis inhibitor and traditionally used to show that a process requires newly synthesized proteins. A possible signal for the cell to release DNA could be H_2_O_2_ induced DNA damage. Treatment with DNA damaging agents like UV-light and mitomycin C also triggered the release of DNA under anaerobic conditions (Itzek et al., 2011). Further mechanistic studies showed that the release process seems to be a lytic process (Xu and Kreth, 2013), but different from the complete cell lysis observed for the DNA release in pneumococci and enterococci since extracellular DNA release can be induced by H_2_O_2_ without any obvious bacterial cell lysis (Itzek et al., 2011). A connection of H_2_O_2_ dependent lysis and extracellular DNA release with competence development is not surprising considering the already described examples for other streptococci. *S. gordonii* expresses the murein hydrolase, LytF. In fact, *lytF* is only expressed during competence because its expression is under the control of the competence stimulating peptide CSP. This observation is reminiscent of the expression of CbpD in *S. pneumoniae* and LytF has been proposed as functional analog of CbpD. DNA transfer experiments relying on LytF dependent cell lysis and subsequent DNA uptake by *S. gordonii* showed that most cells are protected from the muralytic activity of LytF (Berg et al., 2012). This is in agreement with the observed lysis resistant population in S. gordonii after H_2_O_2_ addition (Itzek et al., 2011). A close association, however, of H_2_O_2_ induced release of DNA and competence development is evident since cells grown under H_2_O_2_ producing conditions are also induced for competence development (Itzek et al., 2011). Interestingly, competence development in *S. pneumoniae* can be initiated by mitomycin C induced DNA damage, which also leads to the release of DNA similar to what was reported for *S. gordonii* (Prudhomme et al., 2006).

Combining the experimental findings in *S. pneumoniae* and *S. gordonii* a clearer picture emerges about the association and ecological advantage of H_2_O_2_ induced DNA release with the adaptation of oral streptococci to stress. *S. gordonii* and probably other H_2_O_2_ producing oral streptococci release DNA into the environment as a consequence of DNA damage. This pool of released DNA likely contains mutations in various genes because of the DNA damage. If such mutated DNA is taken up and integrated into the chromosome, the transformation event would lead to a bacterium able to grow and out compete bacteria without the respective mutation under selective conditions or as a template for the repair of stress-induced DNA damage.

## Limitations of horizontal gene transfer

The successful transfer of a new genetic trait is dependent on several events. In the case of eDNA, the genetic material needs to persist in the environment long enough to be taken up by a competent bacterium. The eDNA integrity and persistence is compromised by host and bacterial derived extracellular nucleases (Kishi et al., 2001; Palmer et al., 2012). Once taken up by competent bacteria, eDNA is subject to cellular defense mechanism which have evolved to prevent the invasion and incorporation of potentially harmful DNA. Restriction modification systems are a well-characterized and common bacterial defense system that methylates genomic DNA at specific target sequences. These sequences are also recognized and cleaved by cognate restriction enzymes when they are not methylated, which will likely be the case on newly acquired, foreign DNA that is taken up by the bacterium, thus preventing integration into the chromosome (Bickle and Kruger, 1993). Another important defense mechanism is the CRISPR-Cas system (clustered regularly interspaced short palindromic repeats), which is considered a bacterial immune system. It is an adaptive and inheritable system which recognizes and destroys foreign DNA therefore preventing infection by bacteriophages, transposons and plasmids. It is an RNA based protection mechanism, which stores parts of the DNA of previously encountered bacteriophage, transposons and plasmids in the CRISPR chromosomal locus. The cell is therefore able to prevent potential harmful DNA of integrating into the chromosome by RNA interference using the stored information of the CRISPR (Gasiunas et al., 2014). In addition, an important limitation is the selective pressure for the respective new genetic trait. If there is no advantage gained by the host bacterium to keep the acquired DNA there will be no selective pressure to maintain it.

## Conclusions

The increasing amount of evidence for HGT in the human oral cavity shows that these processes are important in the adaptability of the oral community. The nature of some of the evolutionary strategies involving HGT is much more complex than simple acquisition of DNA released from dead cells or acquisition of a plasmid or transposon from a donor member of the oral community. More evidence for this is found in oral metagenomes; it has recently been found that the incidence of CRISPRs and the numbers of MGEs associated with oral cavity derived metagenomes is far more than in the GI tract of man (Zhang et al., 2013). The pervasiveness of HGT and the incredibly large number of MGEs found in oral bacteria is arguably a result of the oral environment or at the very least is influenced and selected by the conditions found within it. In other words, if the oral bacterial consortium requires a specific gene, for example conferring antibiotic resistance, it is most-likely already present and only needs to be acquired by the various mechanisms of HGT discussed.

### Conflict of interest statement

The authors declare that the research was conducted in the absence of any commercial or financial relationships that could be construed as a potential conflict of interest.
